# Human Exposure following *Mycobacterium tuberculosis* Infection of Multiple Animal Species in a Metropolitan Zoo

**DOI:** 10.3201/eid0811.020302

**Published:** 2002-11

**Authors:** Peter Oh, Reuben Granich, Jim Scott, Ben Sun, Michael Joseph, Cynthia Stringfield, Susan Thisdell, Jothan Staley, Donna Workman-Malcolm, Lee Borenstein, Eleanor Lehnkering, Patrick Ryan, Jeanne Soukup, Annette Nitta, Jennifer Flood

**Affiliations:** * California Department of Health Services, Berkeley, California, USA; † Centers for Disease Control and Prevention, Atlanta, Georgia, USA; ‡ California Department of Health Services, Sacramento, California, USA; § Los Angeles Zoo, Los Angeles, California, USA; ¶ City of Los Angeles Occupational Health Services Division, Los Angeles, California, USA; # Los Angeles County Department of Health Services, Los Angeles, California, USA

**Keywords:** *Mycobacterium tuberculosis*, outbreaks, animals, zoo, epizootic, zoonoses

## Abstract

From 1997 to 2000, *Mycobacterium tuberculosis* was diagnosed in two Asian elephants (*Elephas maximus*), three Rocky Mountain goats (*Oreamnos americanus*), and one black rhinoceros (*Diceros bicornis*) in the Los Angeles Zoo. DNA fingerprint patterns suggested recent transmission. An investigation found no active cases of tuberculosis in humans; however, tuberculin skin-test conversions in humans were associated with training elephants and attending an elephant necropsy.

Outbreaks of *Mycobacterium tuberculosis* have been documented in environments such as hospitals, schools, factories, homeless shelters, and prisons (1–5). In more unconventional settings, such as circuses and exotic animal facilities, outbreaks pose unique tuberculosis (TB) control challenges because transmission may involve animals as well as humans (6,7). Zoos are a particular public health concern because of the close contact between TB-susceptible animals and humans, specifically animal handlers and visitors to the facility or exhibit. Infection and disease related to *M. tuberculosis* have been reported for a variety of species ranging from birds to primates (8–10). Although evidence for human-to-animal transmission of *M. tuberculosis* has been described (11), little documentation of zoonotic transmission to humans exists (7). We describe the first reported multispecies epizootic of genotypically identical strains of *M. tuberculosis* in a zoo and the results of an investigation of exposed zoo employees.

## Synopsis of Animal TB Cases

From 1997 to 2000, *M. tuberculosis* was identified in six animals at the Los Angeles Zoo. In March 1997, an Asian elephant (*Elephas maximus*) (elephant 1) died of salmonellosis. During the necropsy, pulmonary lesions were discovered, and a lymph node specimen showed *M. tuberculosis*. In April 1997, a positive trunk wash culture of *M. tuberculosis* was obtained from a second Asian elephant (elephant 2), which had resided in the same barn as elephant 1. In July 1998, a Rocky Mountain goat (*Oreamnos americanus*) (goat 1) suffered deterioration associated with worsening pneumonia; the pathologic examination was consistent with TB, and culture confirmed *M. tuberculosis.* Tuberculin skin tests of two cohabiting goats (goats 2 and 3) were positive, but their cultures were negative*.* In August 1998, a black rhinoceros (*Diceros bicornis*) had a positive *M. tuberculosis* culture. In February 2000, routine chest radiographs of goats 2 and 3 showed abnormalities consistent with TB. We isolated *M. tuberculosis* from both animals.

## Veterinary Epidemiologic Investigation

We examined medical and location histories of the affected animals as well as handling practices, health-care procedures, and other means of potential exposure to *M. tuberculosis.* An epidemiologic link was defined as documented exposure to an infectious human or animal with TB. We conducted an infection control assessment of the animal compounds and health-care facilities and measured air flow in the compounds by smoke testing (12).

## Examination of Animal Isolates

Elephant isolates (e.g., trunk washes) were obtained according to United States Department of Agriculture guidelines (13). We used saline nasal washes to gather rhinoceros isolates and tracheal washes to gather isolates from goats, as well as specimens for pathologic examination. The Los Angeles County Public Health Laboratory performed restriction fragment length polymorphism (RFLP) analyses on the isolates. Southern blots of *Pvu*II–restricted chromosomal DNA were run in 1% agarose gels, probed with a DNA fragment corresponding to IS*6110*, and detected by chemiluminescence (14).

### Employee TB Screening

Medical records of zoo employees were reviewed for evidence of TB symptoms (i.e., persistent cough, hemoptysis, night sweats, difficulty in breathing, and weight loss), chest radiograph information, and tuberculin skin-test results. In addition, a list of current and former employees was confidentially matched against reported TB cases in the California state registry from 1985 to 2000 (15). During an annual occupational health screening in June 2000, employees participated in TB symptom reviews and received tuberculin skin tests; they also completed a questionnaire on medical history, job type, and history of contact with the infected animals.

### Tuberculin Skin-Test Conversion Categories and Statistical Analyses

A positive tuberculin skin test was defined with a documented induration of >5 mm. We categorized employees with positive tuberculin skin tests as true, probable, or possible converters or as nonconverters. True converters were patients with a negative two-step test (within a 3-week period), followed by an increase in induration of >10 mm within 2 years. Probable converters had no two-step test but had either an induration increase of >10 mm within a 2-year period or two negative (<5 mm) results within 1 year followed by a positive result of >10 mm. Possible converters had an initial negative result followed by a positive test. Nonconverters were patients with positive tuberculin skin tests who did not fit these conversion categories (e.g., one positive tuberculin skin test without a previous test). The questionnaire responses of converters (true, probable, and possible combined) were compared to those of employees with negative tuberculin skin tests.

Relative risk (RR) ratios were calculated by chi-square or Fisher’s exact test by using Epi Info 6 (Centers for Disease Control and Prevention, Atlanta, GA). Statistical significance was considered to be p<0.05.

## Epidemiologic Findings and Genotyping of Animal Isolates

Both elephants with TB had resided at the same exotic animal facility in the United States before arriving at the Los Angeles Zoo in 1994. In 1997, *M. tuberculosis* was found in four other elephants at the exotic animal facility; however, the RFLP pattern differed from that of elephants 1 and 2 (unpub. data). The only documented epidemiologic links among the affected animals were between the two elephants and among the three goats. No common contact outside the animal compounds and no contact with an infectious human was found to account for TB transmission among multiple species.

Standard operating procedures at the zoo included guidelines for animal quarantine and the use of N95 respirators during medical procedures. The elephant compound was 27 m from the rhinoceros compound, and the goat compound was 90 m from both. Smoke tests of the animal compounds showed adequate air movement of 0.3–0.9 m/s and winds of 4.8–8.0 km/hr in ambient conditions.

RFLP analysis showed that five of six animal isolates shared an identical 13-band IS*6110* pattern (Figure). The isolate of goat 3 differed by one additional band.

**Figure:**
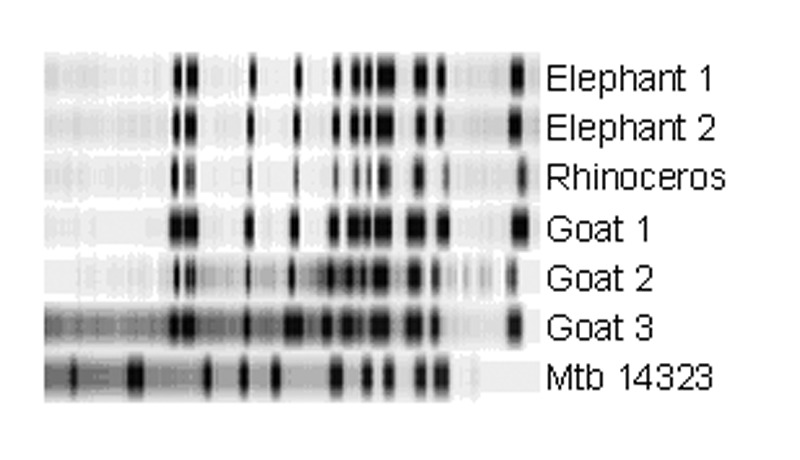
IS*6110* restriction fragment length polymorphism results of the six animal isolates and the *Mycobacterium tuberculosis* reference strain Mtb14323. Molecular weights of IS*6110*-containing *Pvu*II fragments of the reference strain are approximately 17, 7.4, 7.1, 4.5, 3.6, 3.1, 2.1, 1.9, 1.7, 1.5, and 1.4 kb.

## Employee Screening Findings

No active TB cases in humans were found during employee TB screening, medical records review, or query of the state case registry. Of 1,088 employees, no matches were identified in the database of cases reported from 1985 to 2000.

Of 336 employees screened for TB during this investigation, 332 (99%) completed the questionnaire, and 307 (91%) had a tuberculin skin test. Of the 323 employees who responded to the job category question, most were veterinarians or animal keepers (112 [35%]) and administrative staff (73 [23%]); other job categories included maintenance workers (38 [12%]), custodians (29 [9%]), and groundskeepers (24 [7%]). Sixty-two percent of the animal handlers were Caucasian, and 61% of the groundskeepers were Hispanic. Sixty percent of all employees reported contact with animals.

Of the 307 employees who had tuberculin skin tests, 55 (18%) reported a positive result. Of these, none reported TB symptoms, and chest radiographs showed no abnormalities suggesting active TB. Persons with positive tuberculin skin tests were more likely than persons with negative tests to be men (RR 3.7, 95% confidence interval [CI] 2.0% to 6.8%), groundskeepers (RR 2.6, 95% CI 1.5% to 4.7%), construction workers (RR 2.5, 95% CI 1.3% to 4.8%), or attendees at the elephant necropsy (RR 2.9, 95% CI 1.5% to 5.5%). However, animal caretaking and animal contact were not associated with a positive tuberculin skin test. In this group of employees, we found no true converters, 10 (18%) probable converters, 5 (9%) possible converters, and 40 (73%) nonconverters.

Risk factors for tuberculin skin-test conversion are described in the table. Employees reporting attendance at elephant 1’s necropsy were more likely to have documented tuberculin skin-test conversions than those not present (RR 6.3, 95% CI 2.1% to 18.9%). Furthermore, employees who trained elephants were more likely to have tuberculin skin-test conversions than those who did not train elephants (RR 4.1, 95% CI 1.3% to 13.1%). Groundskeepers (n=24) had an increased risk of tuberculin skin-test conversion compared with other job categories (RR 7.1, 95% CI 2.6% to 19.1%). Four of five groundskeepers with tuberculin skin-test conversions were born in the United States; 11 of 14 employees with negative skin tests and none of the 5 groundskeepers with positive tuberculin skin tests (in the nonconverter group) were born in the United States. A lower likelihood of tuberculin skin-test conversion was associated with visiting the animal nursery (RR 0.2, 95% CI 0.0% to 0.7%) and the health center (RR 0.3, 95% CI 0.1% to 0.9%).

**Table:** Relative risks for tuberculin skin-test conversion based on answers reported on employee exposure questionnaires^a^

Characteristics reported in questionnaire	Converters^b^ (%)	TST-negatives^c^ (%)	Risk ratio (95% CI)	p value
Male	13 (87)	112 (45)	7.3 (1.7 to 31.9)	0.002
Ethnicity				
Hispanic	10 (67)	109 (44)	2.4 (0.8 to 6.8)	0.092
White	2 (13)	105 (43)^d^	0.2 (0.1 to 1.0)	0.025
Black	2 (13)	22 (9)	1.5 (0.4 to 6.3)	0.41
Asian	1 (7)	9 (4)	1.8 (0.3 to 12.3)	0.45
U.S.-born	11 (73)	226 (90)	0.4 (0.1 to 1.0)	0.052
BCG vaccine history	0 (0)	15 (7)^e^	0.0 (0.0 to 6.0)^f^	0.40
Contact with any animal	7 (50)^g^	147 (60) ^h^	0.7 (0.2 to 1.9)	0.45
Job type				
Animal care	4 (29) ^g^	91 (37) ^i^	0.7 (0.2 to 2.2)	0.54
Groundskeeping	5 (36) ^g^	14 (6)	7.1 (2.6 to 19.1)	<0.001
Custodial	2 (14) ^g^	19 (8)	1.9 (0.5 to 8.0)	0.31
Maintenance	1 (7)	15 (6)	1.2 (0.2 to 8.5)	0.60
Construction	1 (7)	11 (4)	1.6 (0.2 to11.3)	0.49
Administrative	1 (7)	67 (27)	0.2 (0.0 to 1.7)	0.082
Animal health center exposure	5 (33)	152 (63) ^j^	0.3 (0.1 to 0.9)	0.025
Elephant compound exposure	5 (33)	130 (52)	0.5 (0.2 to 1.4)	0.17
Trained elephants	3 (20)	12 (5)	4.1 (1.3 to 13.1)	0.045
Visited elephants	2 (13)	77 (31)	0.4 (0.1 to 1.6)	0.12
Attended necropsy	3 (20)	7 (3)	6.3 (2.1 to 18.9)	0.014
Goat compound exposure	3 (20)	72 (29)	0.6 (0.2 to 2.2)	0.35
Rhino compound exposure	2 (13)	82 (33)	0.3 (0.1 to 1.5)	0.097
Animal nursery exposure	2 (13)	124 (50) ^i^	0.2 (0.0 to 0.7)	0.007

^a^TST, tuberculin skin test; CI, confidence interval. ^b^Employee with a positive TST in any of the converter categories as described (true, probable, or possible). N=15, except where noted. ^c^N=251, except where noted. ^d^N=245. ^e^N=232. ^f^Odds ratio reported because of a zero value. ^g^N=14. ^h^N=244. ^i^N=248. ^j^N=243.

## Conclusions

Although 55 zoo employees showed evidence of *M. tuberculosis* infection, no person with active TB disease was identified. Given the public’s distance from the animals and the absence of active TB among employees with closer contact with these animals, *M. tuberculosis* was likely not transmitted from humans to animals at this zoo.

The finding that groundskeepers and not animal handlers were associated with a higher risk of tuberculin skin-test conversion was unexpected. Because groundskeepers as a group were more likely to be born outside of the United States than animal keepers, we hypothesized that tuberculin skin-test conversion may have resulted from infections acquired outside of the zoo. However, within this group, only one of five groundskeepers with a tuberculin skin-test conversion was born outside of the country. This finding suggests that a recent exposure may have been responsible for tuberculin skin-test conversion in this occupational category, although small numbers limit the inference.

Genotyping evidence strongly suggested transmission from one species to another, although corroborating epidemiologic evidence of transmission was not discovered. One explanation for transmission is that the elephants may have been exposed to TB at the animal facility in which they resided before their arrival at the zoo. The distances to other animal compounds at the zoo make airborne spread unlikely. In addition, we found no employees with active TB. Since interspecies transmission routes were not found, we suggest that continued vigilance for sources of ongoing transmission is warranted.

Finally, we did discover a significant association between tuberculin skin-test conversion and attending the elephant necropsy and training elephants in the compound. This report emphasizes the importance of adhering to strict infection control measures during large animal necropsies and medical procedures, even when TB is not suspected, because of potentially large bacillary loads.
